# 

**Published:** 1894-03

**Authors:** 


### CONTENTS FOR MARCH, 1894.

ORIGINAL ADDRESS. PAGE.

Annual Address of the President Before the Medical Society of the County of Erie. By JOHN PARMENTER, M. D. .......... 449

ORIGINAL COMMUNICATIONS.

Dysmenorrhea. By ELECTA B. WHIPPLE, A. M., M. D. .......... 457
Observations on Fractures and Their Treatment. By GREGORY DOYLE, M. D .......... 465

TRANSLATION.

What Value has the Microscope in Examinations for Gonorrhea? By G. A. HIMMELSBACH, M. D .......... 471

CLINICAL MEMORANDUM.

White's Operation for Enlarged Prostate .......... 481

ABSTRACT.

The Treatment of Uterine Fibroids .......... 482

EDITORIAL.

The Medical Society of the State of New York .......... 484
Topics of the Month .......... 486

Personal .......... 492

Obituary .......... 494

Book Reviews .......... 494

Literary Notes .......... 511

